# Supplementary material for: Human Cytomegalovirus Modifies Placental Small Extracellular Vesicle Composition to Enhance Infection of Fetal Neural Cells In Vitro
Source: Viruses. 2022 Sep 13;14(9):2030. doi: 10.3390/v14092030 (PMC9501265; doi:10.3390/v14092030)
Supplement: Supplementary file 1 [file viruses-14-02030-s001.zip › Supplementary Material.pdf]

Figure S1: Model system.

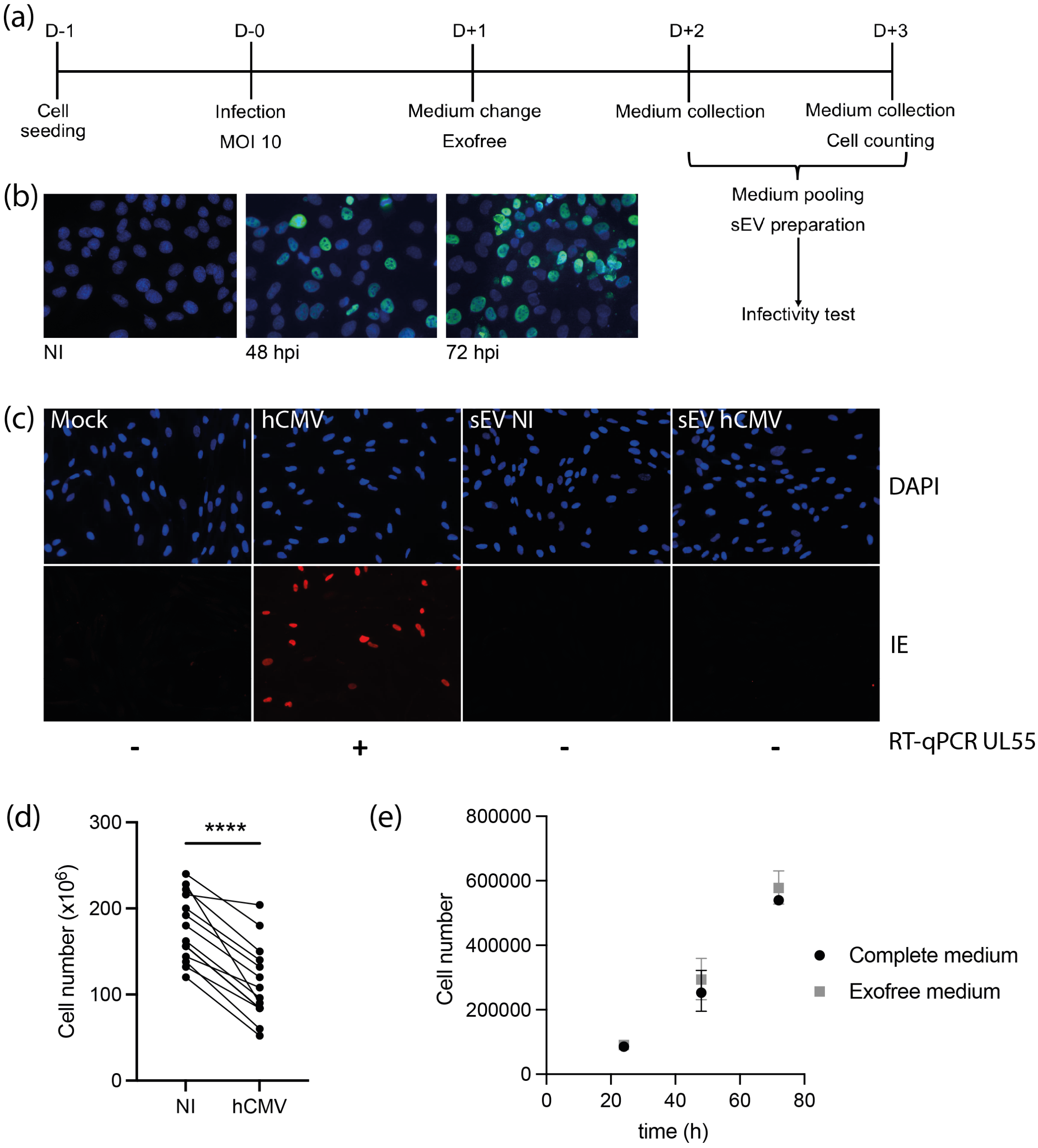

(**a**) Timeline representing the HIPEC seeding, infection, medium renewal and collection before sEV preparation. D: days; MOI: Multiplicity of infection. (**b**) HIPECs are permissive to hCMV infection. Immunofluorescence against Immediate Early 1/2 (IE) antigens was done on HIPECs either non-infected (NI) or at 48 h or 72 h post-infection (hpi). Green: IE; Blue: DAPI; Magnification: 20 x. (**c**) Immunofluorescence realized against hCMV IE1/2 antigens in non-treated MRC5 cells (Mock), or upon incubation during 24 h with either hCMV at MOI 3 (hCMV), sEVs isolated from non-infected (sEV NI) or from hCMV-infected HIPECs (sEV hCMV). Magnification = 20 x. Blue (upper panel): DAPI; red (lower panel): IE. Below images are indicated the results of RT-qPCR realized against hCMV UL55 mRNAs on RNA extracted from MRC5 cells at 48 h post-incubation with hCMV or sEV preparations (+: amplification; -: no

amplification). Data are representative for at least three independent experiments. (**d**) Total cell number counted at the time of sEV preparation, at 72 hpi, for non-infected (NI) or hCMV infected HIPECs. Results are representative of 15 independent experiments. $p<0.0001$ by paired *t*-test. (**e**) Comparison of cell growth between complete and Exofree culture medium, by counting of HIPEC number in 12-well plates in which 10^5 cells were initially seeded. Symbols represent mean ± SEM for three independent experiments. A 2-way ANOVA statistical test indicated no significant difference between complete and Exofree medium culture conditions ($p=0.4414$).

Figure S2: Wide-field transmission electron microscopy.

NI

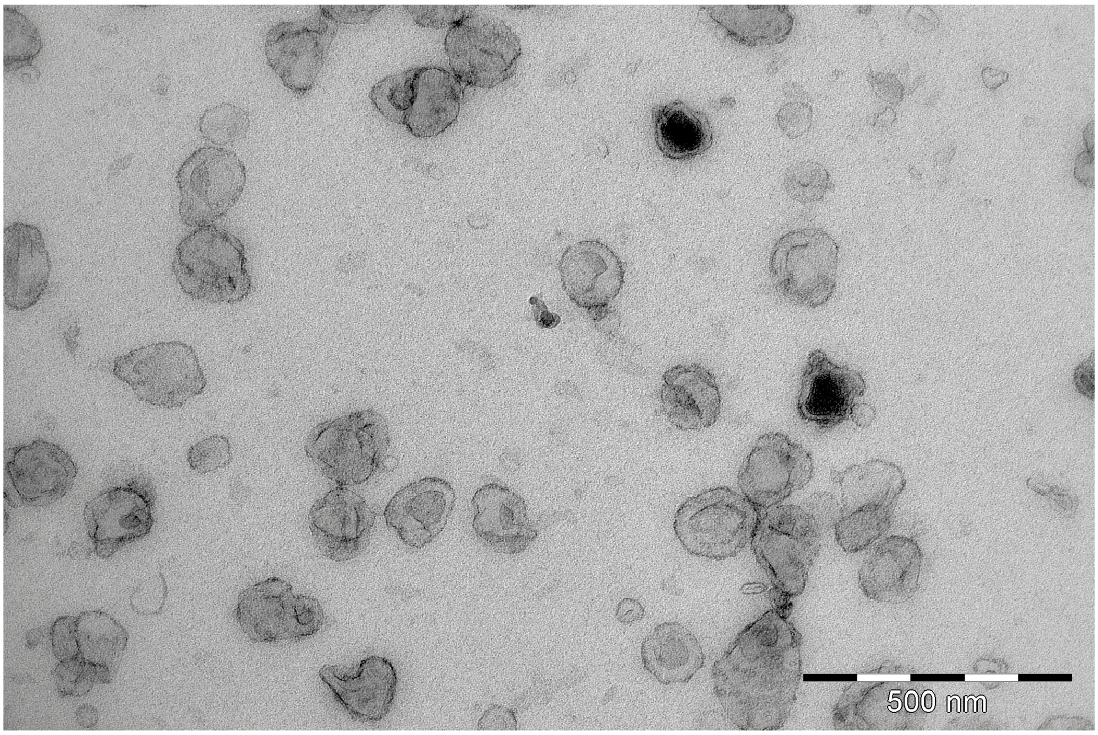

hCMV

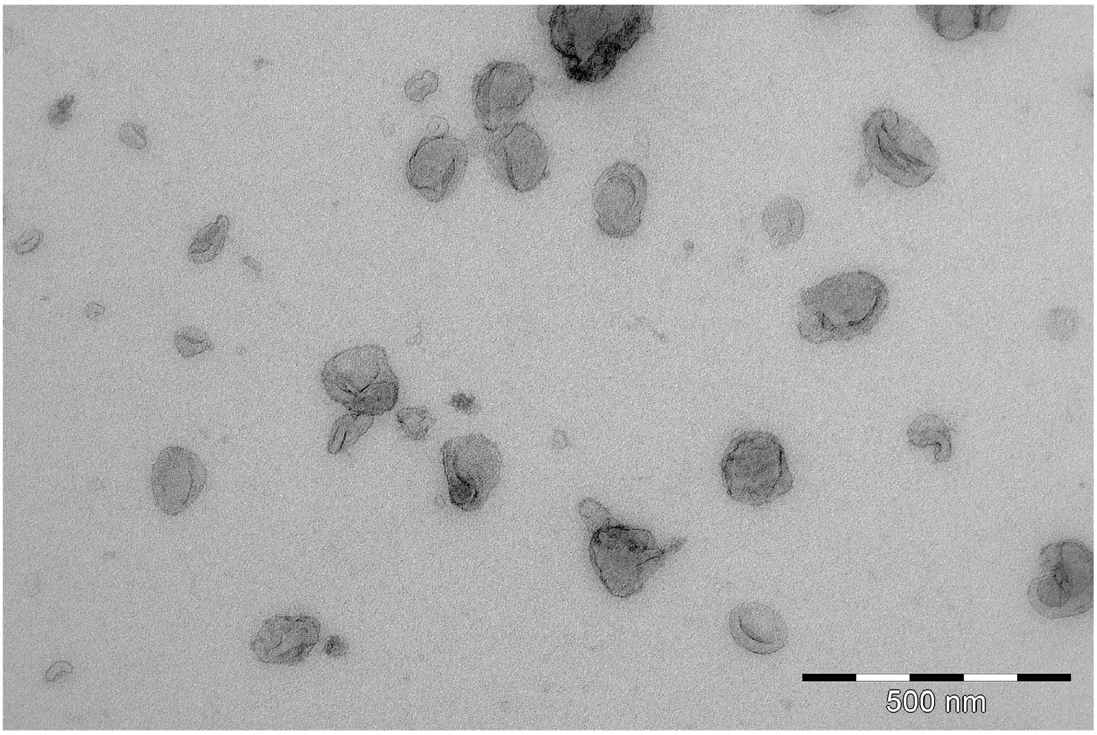

TEM micrographs showing sEV from HIPEC either non-infected (NI; upper panel) or infected (hCMV; lower panel). Scale bar: 500 nm.

Figure S3: Multiplex bead-based flow cytometry comparison of sEV prepared from non-infected of hCMV-infected HIPECs.

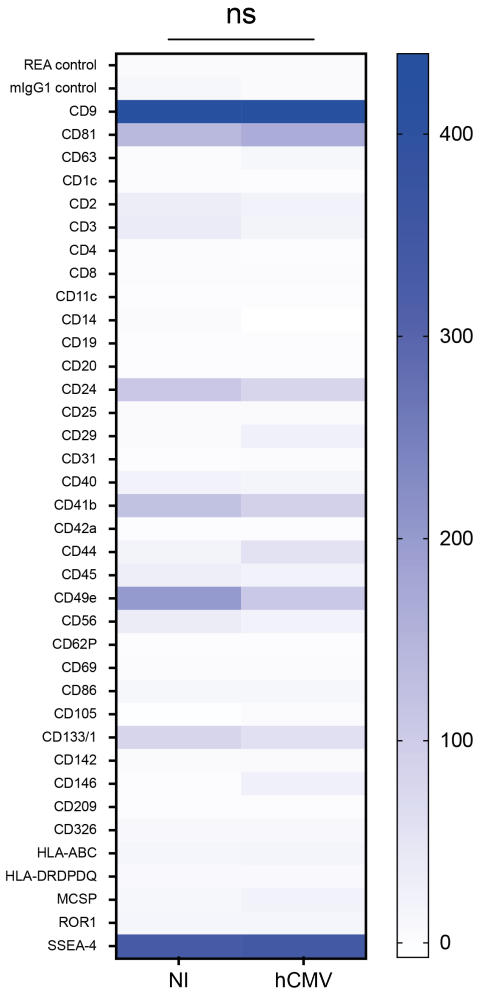

Bead-based multiplex analysis using the MACSPlex Exosome Kit, human (Miltenyi Biotec) were realized on sEV preparations by flow cytometry according to the manufacturer's instructions. This allowed the quantification of 39 different EV markers, distinguishable by flow cytometry by a specific PE and FITC labeling. The MACSQuant Analyzer 10 flow cytometer (Miltenyi Biotec) was used for analysis. The tool MACSQuantify was used to analyze data (v2.11.1746.19438). GraphPad Prism (v8) software was used to perform statistical analysis of the data. The heat-map represents the mean of 3 independent experiments, for different sEV markers indicated on the left column, for sEV prepared from non-infected of hCMV-infected HIPECs. Blue intensity is proportional to the level of expression calculated in Median Fluorescence Intensity, indicated on the right of the heat-map. ns, non-significant by two-way ANOVA.

Figure S4: Wide field immuno-electron microscopy anti CD63.

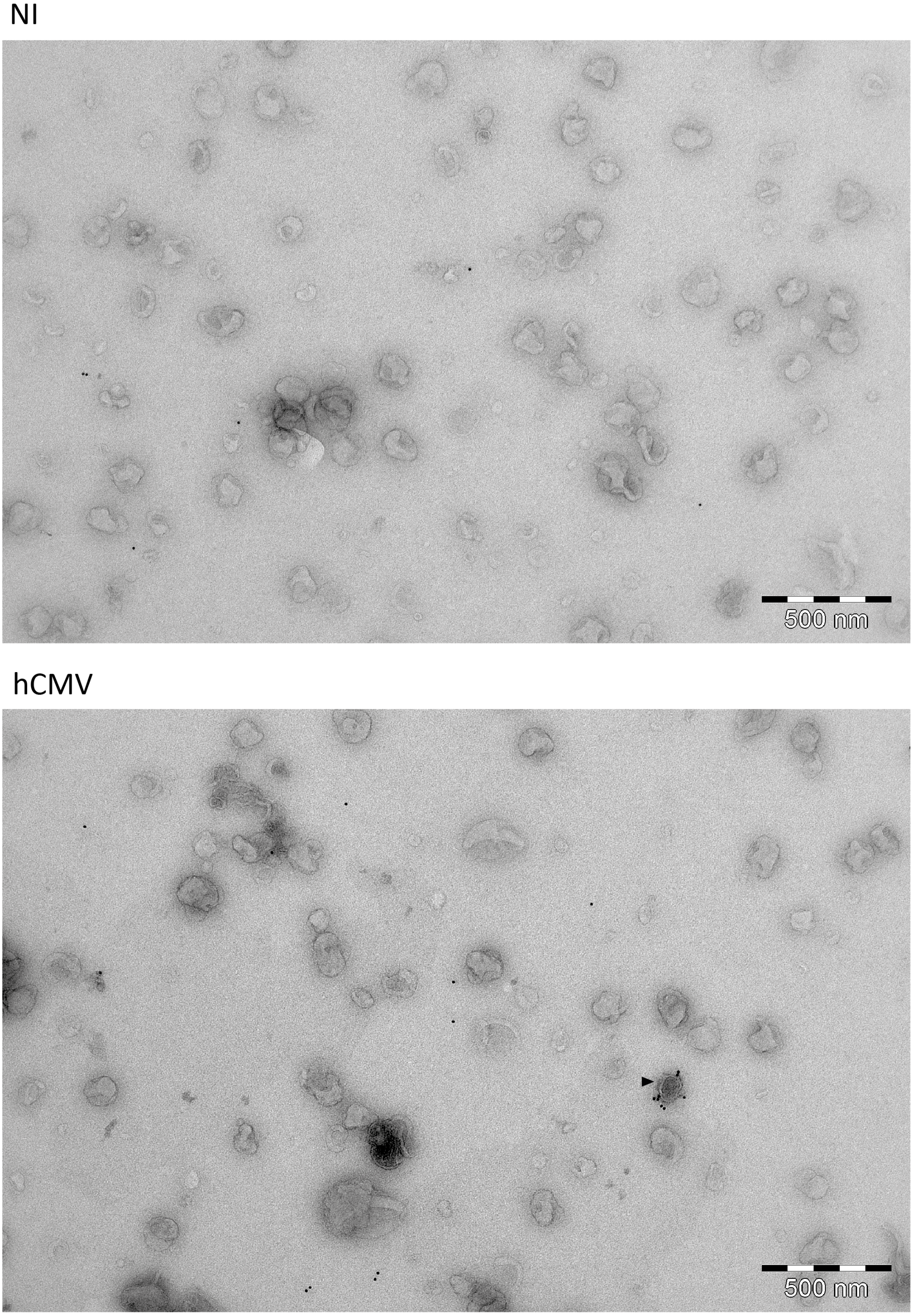

TEM micrographs showing sEV from HIPECs either non-infected (NI; upper panel) or infected (hCMV; lower panel), after immunogold labelling against CD63. Arrow in the lower panel indicates a CD63 positive sEV. Scale bar: 500 nm.

Figure S5: Complement to proteomic analysis.

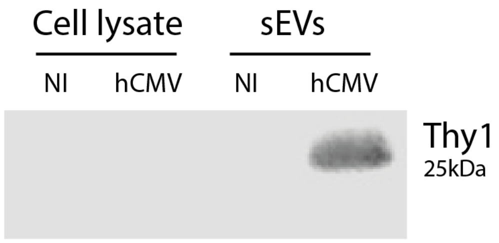

Western-blot showing the enrichment of the cellular Thy-1 protein in sEVs from infected HIPECs (NI: non-infected). This result is representative of three independent experiments.

Figure S6: sEV internalization by MRC5 at 2 h post-incubation.

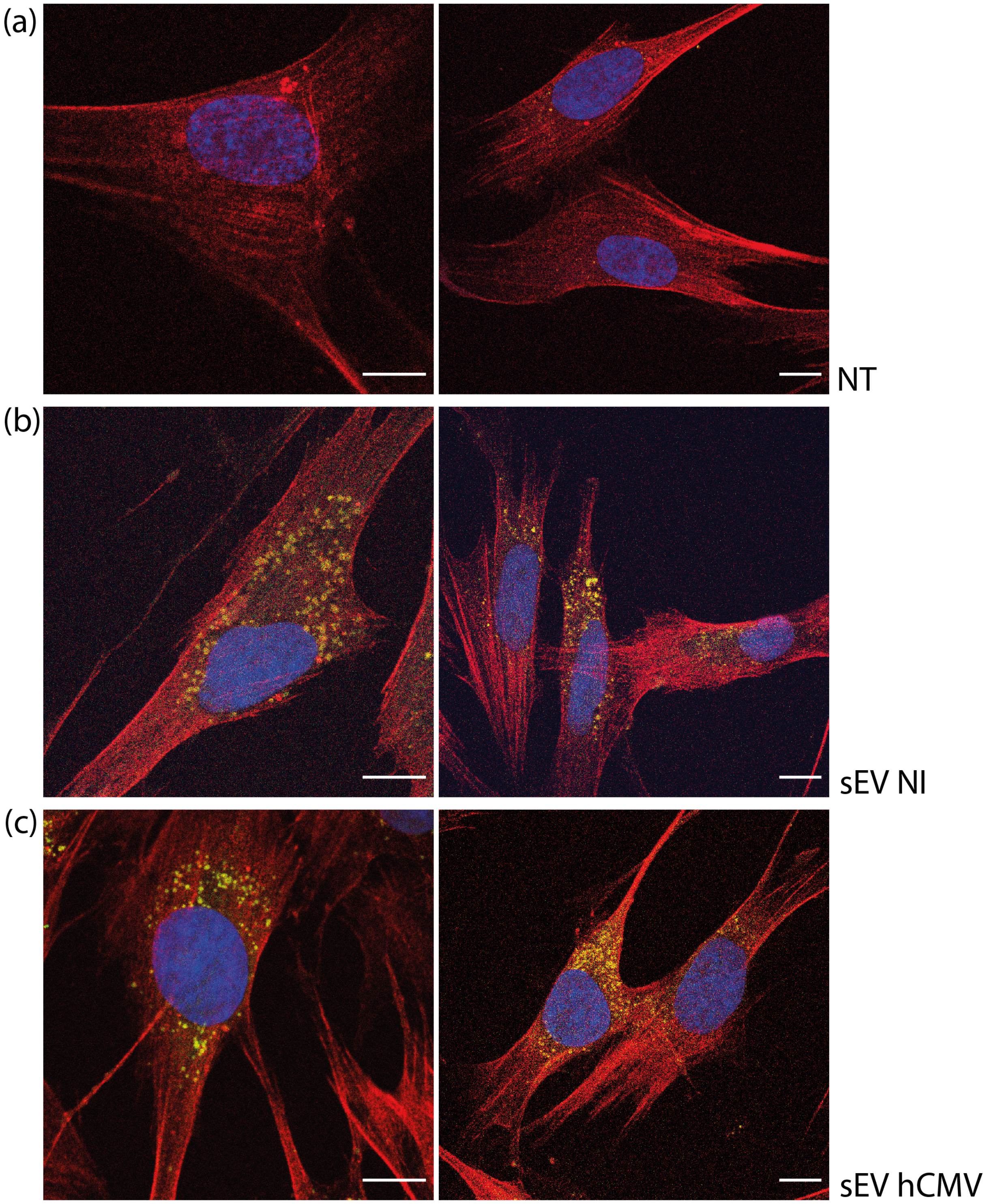

MCR5 cells, observed by confocal fluorescence microcopy, upon 2h of incubation with PKH67 stained sEVs (200 sEVs/cell) prepared from non-infected (panels b), hCMV-infected (panels c) or without sEV incubation (panels a). Green: PKH67 staining of sEV; red: actin staining by phalloidin; blue: DAPI. Scale bar: 10 μm.

Table S1: Excel file.

The mass spectrometry proteomics data have been deposited to the ProteomeXchange Consortium via the PRIDE partner repository with the dataset identifier PXD029146.

Table S2: Bibliography references for establishment of Figure 2d

Protein name	Gene name	Reference	Link
**Capsid scaffolding protein**	UL80	Loveland *et al.* J. Virol. 2007	https://doi.org/10.1128/JVI.01903-06
**Capside smallest protein**	UL48/49	Yu *et al.* J. Virol 2005	https://doi.org/10.1128/JVI.79.2.1327-1332.2005
**DNA polymerase processivity factor**	UL44	Silva *et al.* Virology 2011	https://doi.org/10.1016/j.virol.2011.06.008
**Early phosphoprotein p84**	UL112	Schommartz *et al.* J. Virol. 2017	https://doi.org/10.1128/JVI.00254-17
**Glycoprotein B**	GB / UL55	Isaacson *et al.* J. Virol 2009<br>Halary *et al.* Immunity 2002	https://doi.org/10.1128/JVI.01251-08<br>https://doi.org/10.1016/S1074-7613(02)00447-8
**Glycoprotein H**	UL75	Yurochko et al. J.Virol 1997	https://doi.org/10.1128/jvi.71.7.5051-5059.1997
**Glycoprotein M**	GM	Mach *et al.* J.Virol. 2020<br>Liu *et al.* Sci. Adv. 2021	https://doi.org/10.1128/JVI.79.4.2160-2170.2005<br>https://doi.org/10.1126/sciadv.abf3178
**Glycoprotein N**	UL73	Mach *et al.* J. Virol. 2005<br>Shimamura *et al.* J. Virol. 2006	https://doi.org/10.1128/JVI.79.4.2160-2170.2005<br>https://doi.org/10.1128/JVI.80.9.4591-4600.2006
**Glycoprotein UL16**	UL16	Dunn *et al.* JEM 2003<br>Müller *et al.* PloS Pathog. 2010	https://dx.doi.org/10.1084%2Fjem.20022059<br>https://doi.org/10.1371/journal.ppat.1000723
**IE1**	UL123	Mocarski *et al.* PNAS 1993<br>Nevels et al. PNAS 2004	https://dx.doi.org/10.1073%2Fpnas.93.21.11321<br>https://doi.org/10.1073/pnas.0407933101
**IE2**	UL122	Castillo *et al.* J. Virol. 2000<br>Paulus *et al.* Viruses 2009	https://doi.org/10.1128/JVI.74.17.8028-8037.2000<br>https://doi.org/10.3390/v1030760
**IRS1**	IRS1/US22	Child *et al.* J. Virol 2004<br>Marshall *et al.* J. Virol 2009	https://doi.org/10.1128/JVI.78.1.197-205.2004<br>https://doi.org/10.1128/JVI.02489-08
**Large tegument protein deneddylase**	UL48	Kim *et al.* J. Virol. 2016	https://doi.org/10.1128/JVI.02766-15
**Major capsid protein**	UL86	Furhmann *et al. J.* Infect. Dis. 2008	https://doi.org/10.1086/587692
**Nuclear egress protein 1**	UL53	Salm *et al.* J. Virol. 2009	https://doi.org/10.1128/JVI.02441-08
**pp150**	UL32	Bogdanow *et al.* PNAS 2013<br>Tandon *et al.* J. Virol 2008	https://dx.doi.org/10.1073%2Fpnas.1312235110<br>https://doi.org/10.1128/JVI.00533-08
**pp28**	UL99	Silva *et al.* J. Virol. 2003	https://doi.org/10.1128/JVI.77.19.10594-10605.2003
**pp65**	UL83	Cristea *et al.* J. Virol. 2010<br>Tomtishen *et al.* Virol. J. 2012<br>Arcangeletti *et al.* J. Cell Biochem. 2011	https://doi.org/10.1128/JVI.00139-10<br>https://dx.doi.org/10.1186%2F1743-422X-9-22<br>https://doi.org/10.1002/jcb.22928
**pp71**	UL82	Kalejta *et al.* Mol. Cell Biol. 2003<br>Tomtishen *et al.* Virol. J. 2012	https://dx.doi.org/10.1128%2FMCB.23.6.1885-1895.2003<br>https://dx.doi.org/10.1186%2F1743-422X-9-22
**pp85**	UL25	Nobre *et al.* eLife 2019	https://doi.org/10.7554/eLife.49894
**Protein UL88**	UL88	Kumar *et al* . J. Virol. 2020<br>Kalejta MMBR 2008	https://doi.org/10.1128/JVI.00474-20<br>https://doi.org/10.1128/MMBR.00040-07
**RIR1**	UL45	Patrone *et al.* J. Gen. Virol. 2003	https://doi.org/10.1099/vir.0.19452-0
**Tegument protein UL23**	UL23	Feng *et al.* PloS Pathog. 2018<br>Fenge *et al.* Front. Microbiol. 2021	https://doi.org/10.1371/journal.ppat.1006867<br>https://dx.doi.org/10.3389%2Ffmicb.2021.692515
**Protein UL51**	UL51	Borst *et al.* J. Virol 2013	https://doi.org/10.1128/JVI.01955-12
**Tegument protein US22**	US22	Kalejta MMBR 2008	https://doi.org/10.1128/MMBR.00040-07
**Tegument serine/threonine kinase**	UL97	Krosky *et al.* J. Virol 2003<br>Hertel *et al.* PloS Pathog. 2007	https://doi.org/10.1128/JVI.77.2.905-914.2003<br>https://doi.org/10.1371/journal.ppat.0030006
**Tegument UL35**	UL35	Salsman *et al.* J. Virol. 2012<br>Fabits *et al.* Microorganisms 2020	https://doi.org/10.1128/JVI.05442-11<br>https://doi.org/10.3390/microorganisms8060790
**Triplex capsid protein 1**	UL46	Varnum *et al.* J. Virol 2004	https://doi.org/10.1128/JVI.78.20.10960-10966.2004
**Triplex capsid protein 2**	UL85	Varnum *et al.* J. Virol 2004	https://doi.org/10.1128/JVI.78.20.10960-10966.2004
**TRS1**	TRS1	Child *et al.* J. Virol 2004<br>Adamo *et al.* J. Virol 2004	https://doi.org/10.1128/JVI.78.1.197-205.2004<br>https://doi.org/10.1128/JVI.78.19.10221-10229.2004
**UL37 immediate early glycoprotein**	UL37	McCormick *et al.* J. Virol. 2003	https://doi.org/10.1128/JVI.77.1.631-641.2003
